# Clinical observation of magnesium aluminum carbonate combined with rabeprazole-based triple therapy in the treatment of helicobacter pylori-positive gastric ulcer associated with hemorrhage

**DOI:** 10.12669/pjms.38.5.5174

**Published:** 2022

**Authors:** Peng-zhe Zhou, Lei Gao, Li-wei Wang, Ying-fu Zhang, Wei-li Song, Ying-xia Hao

**Affiliations:** 1 Peng-zhe Zhou, Department of Gastroenterology, Baoding First Central Hospital, Hebei, Baoding, 071000, P. R. China; 2 Lei Gao, Department of Gastroenterology, Baoding First Central Hospital, Hebei, Baoding, 071000, P. R. China; 3 Li-wei Wang, Department of Gastroenterology, Baoding First Central Hospital, Hebei, Baoding, 071000, P. R. China; 4 Ying-fu Zhang, Department of Gastroenterology, Baoding First Central Hospital, Hebei, Baoding, 071000, P. R. China; 5 Wei-li Song, Department of Gastroenterology, Baoding First Central Hospital, Hebei, Baoding, 071000, P. R. China; 6 Ying-xia Hao, Department of Gastroenterology, Baoding First Central Hospital, Hebei, Baoding, 071000, P. R. China

**Keywords:** Magnesium aluminum carbonate, Rabeprazole-based triple therapy, Helicobacter pylori positive, Gastric ulcer, Gastrointestinal hemorrhage, Treatment

## Abstract

**Objectives:**

To evaluate the clinical effect of magnesium aluminum carbonate combined with rabeprazole-based triple therapy in the treatment of patients with Helicobacter pylori-positive gastric ulcer associated with hemorrhage.

**Methods:**

A total of 80 patients with Helicobacter pylori-positive gastric ulcer associated with hemorrhage admitted to the Baoding First Central Hospital from January 2019 to December 2020 were selected and randomly divided into two groups, with 40 cases in each group. The control group were given rabeprazole-based triple therapy, while the experimental group were treated with magnesium aluminum carbonate on the basis of the control group. The changes of symptoms and signs such as abdominal pain, abdominal distension, nausea, vomiting and hematochezia were compared between the two groups before and after treatment. Serological changes of the gastric mucosal microenvironment, such as the serum levels of extracellular regulatory protein kinase (ERK), superoxide dismutase (SOD) and epidermal growth factor receptor (EGFR), were compared between the two groups. Moreover, the differences in the results of gastroscopy between the two groups before and after treatment were compared and analyzed.

**Results:**

The scores of gastrointestinal symptoms in the experimental group after treatment were significantly improved compared with the control group (p=0.00). The levels of ERK and EGFR in the experimental group were significantly lower than those in the control group (ERK, p=0.01; EGRF, p=0.00), while the level of SOD was significantly increased (p=0.02). After treatment, the total effective rate of ulcer healing in the experimental group was 82.5%, which was significantly better than 60% in the control group (p=0.03). After treatment, moderate to severe gastric mucosal inflammation in the experimental group decreased to 10%, significantly better than that in the control group (decreased to 30%) (p=0.03).

**Conclusion:**

Magnesium aluminum carbonate combined with rabeprazole-based triple therapy is preferred for the treatment of patients with Helicobacter pylori-positive gastric ulcer associated with hemorrhage. With such a highly effective treatment regimen, the internal environment and blood supply of gastric mucosal cells can be significantly improved, gastric mucosal inflammation and gastrointestinal symptoms can be ameliorated, and the healing of ulcer surfaces can be accelerated.

## INTRODUCTION

Gastric ulcer, as one of the most common chronic digestive system diseases, can be attributed to the inflammatory and necrotic lesions of the gastric mucosa caused by various pathogenic factors acting on the gastric mucosa.^1^,^2^ Patients suffering from gastric ulcer have clinical manifestations as upper abdominal pain, which, if not treated in time, will lead to hemorrhage, gastric perforation and even gastric cancer.^3^ The pathophysiological mechanism of gastric ulcer is the imbalance between gastric mucosal self-defense-repair factors and gastric mucosal damage factors.^4^

It is currently considered that non-steroidal anti-inflammatory drugs^5^ and Helicobacter pylori (Hp) are the key factors leading to the occurrence and development of gastric ulcer.^6^ Among them, Hp infection contributes to more than 70% of patients with gastric ulcer, as well as gastric ulcer hemorrhage.^7^ In view of this, eradication of Hp is an important method for the treatment of Hp-positive gastric ulcer.^8^ Proton pump inhibitors and antibiotics are currently preferred in clinical practice and have achieved certain clinical efficacy. However, gastric ulcer is a chronic disease, above-mentioned anti-HP treatment alone cannot achieve satisfactory outcomes. It has been shown in animal experiments^9^ that aluminum-magnesium mixture has been reported as having a very important role in repairing acid-induced gastric barrier dysfunction and accelerating the repair of acid-affected tissues.

Meta-analysis showed^10^ that the combination of proton pump inhibitors (PPI) and aluminium-magnesium mixture is conducive to significantly reducing hemorrhage in patients with gastric ulcers. Based on the above viewpoints, in this study, aluminum magnesium carbonate combined with rabeprazole-based triple therapy was utilized to treat patients with Hp-positive gastric ulcer associated with hemorrhage, and certain clinical effects were achieved.

## METHODS

A total of 80 patients with Hp-positive gastric ulcer associated with hemorrhage admitted to the Baoding First Central Hospital from January 2019 to December 2020 were selected and randomly divided into two groups: the control group and the experimental group, with 40 cases in each group. In the experimental group, there were 21 males and 19 females, aged 37-75 years old, with an average of 54.00±14.48 years old; While in the control group, there were 24 males and 16 females, aged 42-76 years old, with an average of 56.96±10.98 years old. No significant difference was observed in the comparison of the general data between the two groups, which was comparable between the groups (Table-I).

**Table I:** Comparative analysis of general data between the experimental group and the control group (χ¯±S) n=40.

Indicators	Experimental group	Control group	t/χ^2^	P
Age (years old)	54.00±14.48	56.96±10.98	1.03	0.31
Male (%)	21	24	0.46	0.50
Course of disease before treatment (years)	3.25±0.78	3.47±1.02	1.08	0.27
Smoking (%)	29	32	0.62	0.43
Drinking (%)	28	23	1.35	0.24
Ulcer diameter (cm)	1.74±0.27	1.68±0.31	0.92	0.36

*P>0.05*.

### Ethical approval:

The study was approved by the Institutional Ethics Committee of Baoding First Central Hospital on March 11, 2019 (No. [2019]037), and written informed consent was obtained from all participants.

### Inclusion criteria:

- Patients meeting the diagnostic criteria for gastric ulcer with hemorrhage^11^;
- Patients aged ≥ 18 years;
- Patients with active gastric ulcer confirmed by gastroscopy biopsy and pathological examination;
- Patients diagnosed with Hp infection by C13 breath test or rapid urinase test of gastric mucosa biopsy;
- Patients with gastrointestinal symptoms such as abdominal pain, abdominal discomfort, and abdominal tenderness;
- Patients who have not received relevant treatment within one month before enrollment;
- Patients with favorable treatment compliance who have given informed consent to this study and signed informed consent.

### Exclusion criteria:

- Patients with insufficiency of vital organs;
- Patients with other digestive diseases at the same time;
- Women in pregnancy and lactation period or patients who are allergic to the drugs involved in the study;
- Patients with gastrointestinal perforation, obstruction and malignant lesions;
- Patients with diseases affecting the results of the study, such as other malignant tumors or autoimmune diseases;
- Patients who have recently taken relevant drugs such as immunosuppressants to affect the study.

Patients in both groups were given general treatment, and and gastric motility drugs were given to those with nausea and vomiting. Patients in the control group were given rabeprazole-based triple therapy: clarithromycin 500 mg orally, bid,^12^ amoxicillin 0.5 g orally, tid, rabeprazole 20 mg orally, bid. Patients in the experimental group were treated with 100mg magnesium aluminum carbonate once a day on the basis of the control group. Patients in both groups were given oral administration on an empty stomach in the morning for four weeks.

All patients underwent gastroscopy before treatment and one week after treatment, followed up for six months after treatment, and their changes in various indicators before and after treatment were recorded.

### Observation Indicators:

***Improvement of clinical symptoms:*** Abdominal pain, abdominal distension, nausea, vomiting, and hematochezia were recorded before treatment and one week after treatment in both groups. 0 point for the complete disappearance of the above clinical symptoms after treatment; one point for the significant improvement of the above symptoms; 2 points for the slight improvement of the symptoms; 3 points for no changes;^13^

***Serological indicators of gastric mucosal microenvironment:*** 5ml of peripheral venous blood was drawn from all patients before treatment and in the morning after treatment, and their serum levels of extracellular regulatory protein kinase (ERK), superoxide dismutase (SOD) and epidermal growth factor receptor (EGFR) were detected by enzyme linked immunosorbent assay (ELISA);

***Ulcer healing:*** Gastroscopy was performed before treatment and one week after treatment. The results were judged as: Cured: All ulcers disappeared under gastroscopy; Markedly effective: Ulcer healing area exceeded 80%; Effective: Ulcer healing area up to 50%-80%; Invalid: Ulcer area was reduced by less than 50%,^14^ the total effective rate = cured + markedly effective + effective/100;

***Gastroscopy pathological analysis:*** Gastroscopy was performed before treatment and one week after treatment. 1 to 2 specimens were collected from the inflammation site. HE staining was used to evaluate the degree of inflammation in the specimens, which was divided into: None, mild, moderate, and severe.^15^ Simultaneously, the Hp kit was used to perform a rapid urinary enzyme test on the specimen to detect Hp.

### Statistical Analysis:

All the data were statistically analyzed by SPSS 20.0 software, and the measurement data were expressed as (X̄ ±s). Two independent sample t-test was used for inter-group data analysis, paired t test was used for intra-group data analysis, and χ^2^ was adopted for rate comparison. P<0.05 indicates a statistically significant difference.

## RESULTS

No significant difference was observed in the scores of gastrointestinal symptoms such as abdominal pain, abdominal distension, nausea, vomiting, and hematochezia between the two groups before treatment (p>0.05), and the above scores were lower after treatment than before treatment. However, the experimental group had a more significant improvement in gastrointestinal symptoms and a more significant reduction in the above scores compared with the control group, with a statistically significant difference (p=0.00) (Table-II).

**Table II:** Comparative analysis of symptoms before and after treatment between the two groups (χ¯±S) n=40.

Group		Before treatment*	After treatment	t	P
Abdominal pain	Experimental groupΔ	2.53±0.51	0.38±0.10	26.16	0.00
	Control groupΔ	2.46±0.63	1.05±0.36	12.29	0.00
	t	0.55	11.34	
	p	0.60	0.00		
Nausea and vomiting	Experimental groupΔ	2.84±0.27	0.38±0.04	17.83	0.00
	Control groupΔ	2.78±0.31	1.32±0.11	28.07	0.00
	t	0.92	17.39		
	p	0.36	0.00		
Abdominal distension	Experimental groupΔ	2.83±1.01	1.23±0.21	9.81	0.00
	Control groupΔ	2.77±0.73	2.05±0.54	5.01	0.00
	t	0.30	8.95		
	p	0.76	0.00		
Hematochezia	Experimental groupΔ	2.17±0.36	0.25±0.04	33.52	0.00
	Control groupΔ	2.25±0.17	0.82±0.10	45.86	0.00
	t	1.27	33.47		
	p	0.21	0.00		

* *p* >0.05, Δ*p* <0.05.

After treatment, serum levels of extracellular regulatory protein kinase (ERK) and epidermal growth factor receptor (EGFR) in the experimental group were significantly lower than those in the control group (ERK, p=0.01; EGRF, p=0.00), and the superoxide dismutase level in the experimental group was significantly higher than that in the control group after treatment, with a statistical significance (p=0.02) (Table-III).

**Table III:** Comparative analysis of serum indicators of gastric mucosal microenvironment after treatment between the experimental group and the control group (χ¯±S) n=40.

Group		Before treatment*	After treatmentΔ	t	P
ERK (pg/ml)	Experimental groupΔ	32.85±4.73	24.38±3.76	8.86	0.00
	Control groupΔ	34.47±4.03	29.61±3.97	5.75	0.00
	t	0.11	3.32		
	p	0.92	0.01		
SOD(U/L)	Experimental groupΔ	72.48±13.45	92.66±14.47	6.46	0.00
	Control groupΔ	73.04±12.75	85.35±13.74	4.15	0.00
	t	0.19	2.32		
	p	0.85	0.02		
EGRF (pg/ml)	Experimental groupΔ	1.47±0.35	0.37±0.06	7.74	0.00
	Control groupΔ	1.38±0.44	0.78±0.03	4.73	0.00
	t	0.23	2.96		
	p	0.82	0.00		

* *p*>0.05, Δ*p*<0.05.

The comparative analysis of the treatment effect between the two groups is shown in Table-IV, indicating that the total effective rate of the experimental group and the control group were 60% after treatment. After treatment, the total effective rate of ulcer healing in the experimental group was 82.5%, which was significantly better than 60% in the control group (p=0.03).

**Table IV:** Comparative analysis of ulcer healing after treatment between the two groups (χ¯±S) n=40.

Group	Cured	Markedly effective	Effective	Invalid	Effective rate*
Experimental group	16	12	5	7	33 (82.5%)
Control group	10	8	6	16	24 (60%)
χ^2^					4.94
P					0.03

* *P<0.05*

The detection rates of moderate to severe gastric mucosal inflammation in the experimental group were 87.5% and 82.5%, respectively, with no statistically significant difference (p=0.53). After treatment, moderate to severe gastric mucosal inflammation in the experimental group decreased to 10%, significantly better than that in the control group (decreased to 30%) (p=0.03). No significant difference was found in the negative Hp conversion rate between the two groups after treatment (p=0.07) (Table-V).

**Table V:** Comparative analysis of pathological results of gastroscopy before and after treatment between the two groups (χ¯±S) n=40.

Group		Before treatment (%)	After treatment (%)	χ^2^	P*
Moderate and severe gastric mucosal inflammation (%)	Experimental groupΔ	87.5%(35/40)	10% (4/40)	48.08	0.00
	Control groupΔ	82.5% (33/40)	30% (12/40)	24.44	0.00
	χ^2^	0.39	5.34		
	P*	0.53	0.03		
Hp detection rate (%)	Experimental groupΔ	100% (40/40)	5% (2/40)	72.38	0.00
	Control groupΔ	100% (40/40)	17.5%(7/40)	53.33	0.00
	χ^2^		3.13		
	p		0.07		

* *p*<0.05.

## DISCUSSION

Gastric ulcer, as one of the most common chronic digestive system diseases, can be mainly attributed to the abnormal secretion of gastric acid and pepsin, long-term use of non-steroidal anti-inflammatory drugs, Hp infection and other factors ultimately lead to gastric mucosal damage and weakened defense function.^16^ With the development of society and the changes in people’s lifestyles, patients with gastric ulcer bleeding account for the largest proportion of all gastric ulcer complications.^17^ Its clinical symptoms include melena, hematemesis, and shock reaction in severe cases^18^, which seriously threatens the life and health of patients. Helicobacter pylori (H. pylori) is present in approximately 50% of the world’s population, with infection levels in developing countries reaching more than 70%. Hp selectively colonizes on gastric epithelial cells and produces ammonia ions by secreting urease to hydrolyze urea, which is a common reason for the occurrence of gastric ulcer. Consequently, the key to the treatment of Hp-positive gastric ulcer is to inhibit excessive gastric acid secretion, reduce pepsin activity, and eradicate Hp.

The adverse situation in the treatment of gastric ulcers has been greatly improved by the introduction of proton pump inhibitors (PPI), whose efficacy and safety are well established, into clinical practice. PPI combined with two or more antibiotics boasts a preferable clinical effect in eradicating Hp infection and treating Hp-related peptic ulcer.^19^ However, PPI combined with anti-HP therapy alone cannot achieve satisfactory results for patients with gastric ulcer and bleeding.^20^ Magnesium aluminum carbonate is a commonly used aluminum-magnesium mixture in clinical practice. It has a protective effect on the gastric mucosa and can alleviate more gastrointestinal symptoms by inducing changes in the intestinal microbiota and host immune response.^21^ Inflammation is an important pathogenesis of gastric ulcer. Aluminum-magnesium mixture can inhibit gastric inflammation by reducing the levels of pro-inflammatory cytokines.^22^ EGFR and ERK are crucial pro-inflammatory cytokines. Aluminum-magnesium mixture has been reported to promote gastric protection and ulcer healing via a variety of mechanisms, including increasing serum SOD levels, antagonizing EGFR and ERK, which can significantly reduce gastric mucosal inflammation,^23^ promote ulcer healing and reduce the incidence of re-hemorrhage.

It was confirmed in our study that magnesium aluminum carbonate combined with rabeprazole-based triple therapy in the treatment of patients with Hp-positive gastric ulcers associated with hemorrhage was superior to rabeprazole acid-fast alone or triple therapy of Hp eradication. The scores of gastrointestinal symptoms such as abdominal pain, abdominal distension, nausea, vomiting, and hematochezia in the experimental group after treatment were significantly improved compared with the control group (p=0.00). The levels of ERK and EGFR in the experimental group were significantly lower than those in the control group (ERK, p=0.01; EGRF, p=0.00), while the level of SOD was significantly increased (p=0.02). After treatment, the total effective rate of ulcer healing in the experimental group was 82.5%, which was significantly better than 60% in the control group (p=0.03). The detection rates of moderate to severe gastric mucosal inflammation in the experimental group were 87.5% and 82.5%, respectively, with no statistically significant difference (p=0.53). After treatment, moderate to severe gastric mucosal inflammation in the experimental group decreased to 10%, significantly better than that in the control group (decreased to 30%) (p=0.03). No significant difference was found in the negative Hp conversion rate between the two groups after treatment (p=0.07).

### Limitations of this study:

It includes a small sample size, with short follow-up time. Moreover, nutritional indicators of patients before and after treatment have not been included in this study. In response to this, proactive countermeasures will be taken to further increase the sample size, follow-up time, and analysis of the difference in the long-term incidence of gastric cancer under the two treatment regimens in future studies. At the same time, the recent changes of patients’ nutritional indicators before and after treatment will be increased, so as to evaluate the clinical effect of the treatment regimen more accurately and objectively.

## CONCLUSION

Magnesium aluminum carbonate combined with rabeprazole-based triple therapy is preferred for the treatment of patients with Helicobacter pylori-positive gastric ulcer associated with hemorrhage. With such a highly effective treatment regimen, the internal environment and blood supply of gastric mucosal cells can be significantly improved, gastric mucosal inflammation and gastrointestinal symptoms can be ameliorated, and the healing of ulcer surfaces can be accelerated.

### Authors’ Contributions:

**PZ and** **YH** designed this study and prepared this manuscript, and are responsible and accountable for the accuracy or integrity of the work.

**LG and** **LW** collected and analyzed clinical data.

**YZ and** **WS** significantly revised this manuscript.
